# Anti-migraine agents from an immunological point of view

**DOI:** 10.1186/s12967-020-02681-6

**Published:** 2021-01-06

**Authors:** Mushref Bakri Assas

**Affiliations:** Faculty of Applied Medical Sciences, Department of Medical Laboratory Technology, Immunology Group, King Abdul Aziz University, Jeddah, Saudi Arabia

**Keywords:** Neuroscience, Therapeutic drug monitoring, Adverse drug reactions, Immunology, Infectious disease, Inflammation

## Abstract

The new wave of anti-migraine agents is nothing less than a milestone in our battle to manage this devastating disease. However, concerns have recently increased regarding the safety of these drugs. CGRP, while known as a potent vasodilator, is also a key neural and immune modulator. The roles of CGRP in immune determination, have been studied in depth, with particular focus on its functional significance with respect to common immune challenges i.e., bacterial, viral, fungal and parasitic infections. This review discusses many potential areas of concern in regard to blocking CGRP function and its potential influence on immune milieus during infection, and the risk of adverse effects. Finally, this review recommends specific measures to be taken into consideration when administering anti-CGRP/CGRPR agents.

## Background

The recent wave of migraine preventative drugs targeting calcitonin gene related peptide (CGRP) and its receptor (CGRPR) have been hailed as a breakthrough in our ability to control the debilitating effects of migraines.

The CGRP gene is located on chromosome 11 and encodes a limited family of peptides including calcitonin, αCGRP, and katacalcin [1]. CGRP is an abundant peptide found within many neurons both in the brain and in peripheral nerves, particularly in so-called nociceptive neurons or *pain fibres* [2]. Structurally, CGRP is a 37 amino acid long/3795.405 g/mol neuropeptide with one main receptor through which it exercises its numerous roles. CGRP is present in two forms, αCGRP and βCGRP, with the former being the predominant subtype in humans. CGRP is composed of four domains [3]. Seven NH2 terminus make up the first domain, held together by a disulfide bridge [4].

The CGRPR is a complex molecule of 3 members. A seven-transmembrane-spanning protein called CL receptor (CLR) a member of the B-family of G-protein coupled receptors (GPCRs). CLR is 461 amino acids and is associated with 1 high-affinity receptor to CGRP known as receptor activity modifying protein (RAMP). The three members of RAMP (RAMP1, 2 and 3) have an extracellular amino terminus of approximately 100 amino acids, a single transmembrane section, and a very short carboxyl terminus of approximately 10 amino acids. CGRP typically binds to the CLR-RAMP1 complex, with CLR-RAMP2 and CLR-RAMP3 complexes strictly binding to adrenomedullin a neuropeptide closely related to CGRP and shares many functions with most notably vasodilation. Both molecules of the CGRP receptor form the CGRP binding site and CGRP can cross-link to both CLR and RAMP1. However, it is not clear whether CGRP has specific contacts with both proteins or whether RAMP1 indirectly contributes to the ligand-binding site by modifying the structure of CLR. A lesser known molecule associated with the CGRPR complex is the CGRP-receptor component protein (RCP). This molecule plays an important role in the intracellular cascade generated post CGRP binding and is thought to play a pivotal role in the regulation of CGRP signaling [5]. It is well known that once CGRP binds to its receptor, the CGRP-CGRPR complex is internalised and removed from the cell surface, thereby becoming undetectable by immunohistochemistry [6]. Moreover, CGRP receptors have been shown to function as auto-receptors, regulating CGRP release [7] indicating a tightly regulated method of secretion.

As for its source, C fibres, unmyelinated sensory neurons with the smallest diameter and lowest threshold, as well as Aδ sensory fibers are understood to be the main source of CGRP. These fibres innervate large parts of the body, with extensive perivascular localization, and play dual roles in sensory (nociceptive) and efferent (effector) function [1, 8] The association of CGRP with low threshold sensory nerves highlights its highly rapid role as a crucial member of the sensory nociceptive milieu, facilitating immediate initial triggers and sensing damage/toxins via pain pathways. C fibres express the transient receptor potential vanilloid 1 (TRPV1) receptor on their surface, which we have come to understand is critical in pain processes. TRPV1 activation, subsequently results in CGRP release through an intracellular cascade response, involving calcium as a key intracellular player, that facilitates intracellular cascades that establish the many roles of CGRP [9]. Functionally, CGRP can influence the cardiovascular system being a potent vasodilator [10]. Additionally, CGRP has displayed vascular protective roles suggested by its potential as a therapy for treating cardiovascular diseases, however, the investigation in this specific topic is still ongoing. Interestingly, CGRP is involved in sensory processing and this has been investigated in migraine models. The development of CGRP antagonists helped broaden our understanding of the role CGRP plays in migraine [11, 12].

Migraines are brain disorders affecting over a billion people worldwide [13, 14], with a higher prevalence in women than in men [15]. Interestingly, our understanding of migraines has shifted to a more multidimensional view involving a range of sensory processes with wide implications throughout the central nervous system, with vascular and immunological contributions. Centrally, signs of activation in the hypothalamus, possibly involving thalamus and the limbic system, are present [16]. Indeed, during a migraine attack, regions in the brainstem and the trigemino-vascular pathway are activated eliciting many of the components classically linked to the symptomatology of the migraine attack. Moreover, the trigeminal system is involved in the pain part of the attack and this is believed to be by part exemplified by the release of CGRP in the headache phase of the migraine attack. This mechanism tested by triptan administration (anti-serotonin 5 HT _1D/1B_ receptors) which resulted in the cessation of the pain [17, 18], and It is established now that close to half of all neurons in the trigeminal ganglion express CGRP [19]. Additionally, studies have so far only shown CGRP to be directly linked to the attack, however, other neuronal messengers could, in addition, be involved. Moreover, migraines can be a consequence of the influx of the neuroinflammatory mediator i.e. substance P, which is exacerbated by the release of the vasodilatory CGRP, which acts on the blood vessels and allows further mediators into the target area. The influx is a result of the activation of the trigeminal nociceptors in the meningeal tissues [20], which in turn stimulate the tigemino-vascular afferents which upon activation release CGRP, substance P and neurokinin A [19].

Erenumab (AMG 334) is a fully human monoclonal immunoglobulin IgG2 that inhibits the action of CGRP. Erenumab is the leading anti-CGRPR drug recently approved by the FDA (erenumab: First Global Approval.) for use in the prophylaxis of migraine in adults. In fact, erenumab is one of the first fully human mAbs approved for the treatment of migraine. The first of its kind, erenumab targets CGRP function by antagonizing the CGRPR [21]. This mechanism of action is in contrast to other anti-CGRP drugs (e.g., eptinezumab (ALD403), fremanezumab (TEV-48125), and galcanezumab (LY2951742)) that nullify the peptide itself rather than its receptor [22–27]. Regarding the four anti-migraine drugs of interest, the use of one drug over another would most likely depend on each case. All four had mild to moderate adverse effects with low incidences, importantly, no liver toxicity has been reported, a condition previously reported in other anti-CGRP agents [28]. These drugs are understandably more efficient in migraine cases only because they target CGRP, which appears to be effective in a large portion of the studied population; however, a portion of patients remain non-responsive to these drugs [29].

A role for CGRP and other neuropeptides in immunity was first hypothesized when CGRP + ve nerves were observed to be in close physical proximity to sentinel cells located in the peripheral tissues (i.e., mast cells, macrophages and dendritic cells) [30]. The discovery of neuropeptide receptors on immune cells soon followed, as did functional studies on the effects of neuropeptides on immune cells in vitro [31]. The decades to follow helped develop a clearer understanding of the direct involvement of CGRP in immune function. In summary, functional and physical contacts exist between nerve fibres and a range of immune cells [30, 32, 33], most notably with macrophages [30, 34, 35], mast cells [36–38], dendritic cells [35, 39–42], lymphocytes [43–46], and natural killer (NK) cells [47, 48]. Immunologically, CGRP, now an archetypical neuro-immune connector, involved in host surveillance and immune modulation [30, 49]. CGRP can modulate antigen presentation in dendritic cells [50] and inhibit lipopolysaccharide induction of co-stimulatory signalling via the CD80 and CD28 receptors on dendritic cells and monocytes, thereby affecting T cell functionality [51]. The topic of CGRP and immunity to infections (viral, bacterial or parasitic) has been extensively covered in the past two decades, with numerous studies dedicated to elucidating the many cross-links between the nervous and immune systems at both the cellular and protein levels. This review will discuss the most studied areas relating to CGRP immune-related effects/influences during infection, while highlighting the potential adverse effects that would emerge as a consequence of the systemic blockage of CGRP. Finally, this review will address concerns relating to the doses of the anti-CGRP/CGRPR agents, the limitations we face in scientific research, and the important measures to be considered when administering these preventative agents.

## CGRP prevents viral replication and cross-infectivity

CGRP helps facilitating an effective immune response against a viral infections. While studies remain limited (only few studies have been conducted on the transgenic effects of viral agents as genetic vectors and their secondary influence on CGRP levels), Studies examining the role of CGRP during a viral infection are discussed below.

### Viruses of the nervous system: VZV, herpes, chickenpox and HIV

The intra-nerve *varicella zoster virus* (VZV), a viral agent for both chickenpox and herpes zoster, decreased CGRP gene expression while increasing TRPV1 expression in dorsal root ganglion (DRG) sensory neurons, correlating, as expected, with a decrease in thermal nociception [52], similar findings were discussed in models of HIV-associated neuropathy [53]. Additionally, alterations in the expression of the pro-inflammatory cytokine tumour necrosis factor (TNF) signalling cascade during infection coincided with a decrease in CGRP expression, a phenomenon that fits with our current understanding of the interactions between CGRP and TNF [9].

It is worth noting that the co-localization of viruses, particularly herpes, with CGRP has been previously noted. This close association between the Herpes simplex virus-1 (HSV-1) and sensory neurons carrying CGRP may contribute to the known neurological diseases such as facial palsy, vestibular neuritis or encephalitis. Flowerdew et al. 2013 discussed the increased expression of CGRP and the glial cell line-derived neurotropic factor (GDNF) receptor, a neural marker found in nociceptive nerve types, in human trigeminal nerve samples associated with herpes infection [54]. The commonality of the many types of herpes viruses makes blocking of CGRP a potentially viable and important target for recurrent infections, which are associated with potentially more serious outcomes (including Alzheimer’s disease and dementia) that have been found to positively correlate with herpes [55–57]. Immunologically, HSV-1 infected macrophages treated with CGRP for 12 h were able to elevate levels of IL-1β compared to untreated controls [58]. IL-1β a key inflammatory cytokine which activates macrophages and promotes pro-inflammatory responses towards viral infections, along with inducing T cell proliferation and differentiation.

*Langerhans* cell (LC) mediated-trans-infectivity of CD4 T cells with HIV-1 was ameliorated in the presence of CGRP [59]. CGRP significantly reduces LC intracellular HIV-1 levels through increased HIV-1 degradation and, as a direct consequence, decreases trans-infectivity of CD4 T cells [59]. Furthermore, CGRP stimulated HIV-1 degradation during the early phases of trans-infection of CD4 T cells, an effect mediated by both types of CGRP: alpha and beta. CGRP shifts HIV-1 degradation that happens naturally in the endolysosomes in LC towards the proteasome, inducing therefore by default a proteosomal-type degradation of HIV-1 displaying an ability to modulate HIV-1 degradation in LC, while significantly inhibiting the trans-infectivity process in whole [59]. This inhibition was amplified via the CGRP autocrine/paracrine positive feedback loop [60]. CGRP downregulates C-X-C chemokine receptor type 4 (CXCR4), a surface antigen on LC involved in HIV-1 chemotaxis to LC. In addition, CGRP increases transcription of signal transducer and activator of transcription 4 (STAT4), preventing viral replication [60]. Interestingly, CGRP serum levels were compared between HIV-1 infected humans treated with combination antiretroviral therapy-uninfected or combination antiretroviral therapy-treated patients with primary/acute or chronic HIV-1 infection, as well as from individuals who naturally control HIV-1 infection, namely exposed seronegatives, elite controllers, and long-term non-progressors [61]. CGRP levels decreased in primary/acute or chronic HIV-1 infections while remaining unchanged in exposed seronegatives, elite controllers, and long-term non-progressors. Importantly, CGRP levels correlated positively with CD4 T cell count and negatively with the viral load which suggests that the interactions discussed earlier in this section between CGRP and HIV-1 within the environment of the LC are indeed positively impacting the bodies response to the virus.

## CGRP induces microbiota homeostasis

The influence of certain neuropeptides on bacterial virulence and infectivity is not a recent finding. CGRP, like substance P and neurokinin A, is of similar size and composition to many conventional antimicrobial peptides. CGRP has only a 7 amino acids and 0.26 pI difference in composition when compared to alpha-defensin (HNP-1) (a potent antimicrobial peptide effective against both gram-positive and gram-negative bacteria) [62]. This similarity suggests antimicrobial effects for CGRP similar in nature to HNP-1, if the circumstances require.

The role of CGRP in controlling, manipulating, and regulating bacterial virulence and viability has been discussed in various models. The fact that βCGRP has been found in nociceptive nerve terminals in the skin [63] is interesting [1, 64].

### CGRP and skin homeostasis: promoting friendly bacteria

Indeed, while having little impact on *Staphylococcus aureus* (SA), CGRP increased the virulence of *Staphylococcus epidermidis,* a commensal bacterium that helps regulate the pathogenic SA skin-bacteria communication [65, 66]. Additionally, CGRP induced innate immune responses increasing chemokine IL-8 (CXCL8) production (from macrophages and epithelial cells) resulting in chemotaxis and immune cell recruitment, and antimicrobial protein production (i.e., cathelicidin (LL37)), and, contrastingly, a decrease in beta-defensin-2 (hBD-2) is understood to regulate this inflammation. The increase in virulence correlated negatively with *Staphylococcus epidermidis* penetration process into the target cell affecting its cytotoxic abilities and impacting the internalisation of the bacteria into keratinocytes. CGRP treatment increased the export of the bacterial homolog of human Hsp70 DnaK. Dnak is believed to interfere with the cell wall degrading enzyme autolysin E (AtlE) on the surface of *Staphylococcus epidermidis* responsible for its internalisation into the target cell. The purpose behind this sequence of activations/regulations remains poorly understood. Furthermore, TRPV1 ablation in a *Streptococcus pyogenes* model reduced CGRP levels, improved the prognosis, enhanced wound healing, and improved murine survival rates [67]. Accordingly, a need for a better understanding behind the reason CGRP promotes commensal bacterial potency while simultaneously actively enhancing anti-microbial immune responses could reveal anti-microbial control mechanisms we have yet to appreciate. A better understanding of this phenomenon would help us understand the underlying mechanistic links between the immune system’s response to a certain bacterium while the bacterial potency simultaneously increases in response to CGRP.

In our understanding of the action of CGRP in the presence of a bacterial threat, our knowledge of the role of CGRP in the context of commensal opportunistic and epithelial tissue bound bacteria is limited. While in many models, CGRP favours anti-inflammatory responses, mainly Th2 in nature, it may also be active in processes that eliminate opportunistic threats (e.g., in food poisoning) [68]. In a study by El Karim et al. 2008, the anti-bacterial properties of CGRP mounted against *Escherichia coli*, *Pseudomonas aeruginosa* and *Candida albicans* were examined [62]. All three organisms were treated with CGRP, and the authors discuss for the first time CGRP attaining anti-microbial properties with a clear consequence on bacterial virulence and proliferation while not affecting normal cell viability in their in vitro human oral tissue samples. Albeit the mechanisms leading to the anti-microbial effects were not discussed in this work, findings remain significant.

In the lung, a front exposed to heavy bacterial insults, CGRP significantly decreased leukocyte migration and recruitment and cytokine production in response to the gram-negative aerobic bacteria *Moraxella catarrhalis* [69]. Corroborating with findings discussed earlier, CGRP, along with, but independently of, substance P, inhibited the IL-1β-dependant expression of hBD-2, a neutrophil chemoattractant molecule, on the surface of transfected epithelial cell-line A549 (type II alveolar cells) [69], effectively impairing neutrophil recruitment to the alveolar tissue and promoting anti-inflammatory activity.

### CGRP-TNFα axis

Namai et al. 2018 engineered a genetically modified strain of lactic acid bacteria (gmLAB) with the ability to produce murine rCGRP by introducing a CGRP secretion plasmid into *Lactococcus lactis* NZ9000 [70]. Is this study, a dose-dependent reduction of TNFα expression in murine C57BL/6 lipopolysaccharide (LPS)-stimulated peritoneal macrophages pre-treated with rCGRP (gmLAB) purified from the supernatant of a culture of NZ-CGRP was observed in vitro [70]. The authors demonstrated a potential prophylactic effect of CGRP on TNFα release. Similar results have been demonstrated in different models. The ability of CGRP to downregulate TNFα when challenged by a bacterial agent has been demonstrated in periodontal diseases in which CGRP promotes osteoblast proliferation and reduces the gram-negative *Prophyromonas gingivalis*-induced osteoblast cell apoptosis [71] through the suppression of the cleaving of c-Caspase-3 and c-Caspase-8, two intracellular cascade molecules associated with apoptotic progression. In the same study, CGRP reversed the decrease in osteoblast viability, demonstrating its potential as a prominent element in bone remodelling through its ability to ameliorate the structural decay of osteoblasts resulting from *Prophyromonas gingivalis*-LPS induced cytotoxicity [71].

## CGRP targeting helminths: what we know about the Th1 vs Th2 balance

The involvement of neuropeptides in the immune response to parasites has been studied for decades. In a *Schistosoma mansoni* infection, the increase in CGRP + ve nerve fibre density in response to infection has been associated with mast cell recruitment to the site of infection [72]. This observation indicates an important role for CGRP in mucosal mast cell-derived immunity against *Schistosoma mansoni*. *Leishmania major* skews the affinity of the CGRP receptor complex CLR-RAMP1 towards adrenomedullin as a survival mechanism, preventing the initiation of CGRP derived anti-helminth immune responses [73]. This observation was supported in studies where in the spleen, skin and dorsal root ganglia (L4–L9) of mice susceptible to *Leishmania major* had significantly lower CGRP levels compared to controls [73]. CGRP plays a role in the pathophysiology of cutaneous leishmaniasis, and this role is suggested to be the reason behind the failure of susceptible BALB/c mice (express less CGRP + ve fibres compared to resistant C57BL/6 mice) to mount a proper immune response against *Leishmania* compared to C57BL/6 mice during the first week of infection [73]. CGRP was reduced to extremely low levels in BALB/c mice, which correlated with failure to contain the cutaneous *Leishmania mexicana* infection [74]. Of note, the observed decrease in CGRP + ve nerve fibre density was importantly associated with a decrease in epidermal LC counts at the site of infection [75].

Most helminths survive in a Th1 immune environment. CGRP plays a critical role in countering this by promoting Th2 immune responses. Assas et al. 2016 showed that, in a *Trichuris muris* model, CGRP promotes anti-Th1 processes, significantly downregulating the prominent Th1 cytokine interferon-gamma (IFNγ) [31] attributing to a hostile environment for the infecting helminth. This study followed work published in the previous decade by Levite et al., in which CGRP alone, independent of the antigen model, drove a Th2 immune response by elevating levels of the potent Th2 cytokine IL4 [76]. This is finding remains of great significance to this day, especially with the profound implication it may have on how we understand/explain immunological ‘dogmas’ in the future.

## Conclusion

This review aims not to disqualify nor discourage the use of anti-CGRP/CGRPR anti-migraine agents. In fact, the mere value of it medically overrides all short-term concerns, if any, and have undoubtedly changed the lives of millions of people around the globe. However, this review attempts to scientifically foresee areas of potential concern from an immunological point of view relating to long-term adverse effects. This review is the first to dissect the most prominent features of CGRP involvement in immunity against pathogens highlighting potential clinical implications as a consequence of entirely blocking its functions. The raised concerns do not apply less to single dose treatment but more to long-term treatment of chronic migraine.

For any new drug, its application on different experimental models helps expand our knowledge regarding its efficacy and potential areas of concern, and while animal models for migraines are now in use [77], the lack of proper animal research on the efficacy and safety of these drugs is of concern. For decades, volumes of valuable research data in mouse models have uncovered many roles for CGRP in the immune system and testing these anti-migraine agents on the same models is logically important. However, we are failing to test the current drugs on the same models for various reasons. For example, the current anti-CGRPR drugs in humans when compared to the currently approved anti-CGRPR drug for research purposes (CGRP8-37, half-life of 30 min, in murine-based studies) are much more advanced, and no equivalent can be found for use in murine models limiting our ability to evaluate their effects in vivo in established models of research. This limitation is acknowledged in the reports produced by the EMA. In the EMA report on erenumab, they state, ‘There are no animal migraine models. Therefore, all evidence for a therapeutic effect is derived from clinical data’ (https://www.ema.europa.eu/en/documents/assessment-report/aimovig-epar-public-assessment-report_en.pdf). The doses of the anti-CGRP/CGRPR drugs are striking at first glance due to their extremely high concentration in comparison to physiological levels of CGRP in vivo. Moreover, these drugs have a relatively long half-life (between 28 and 39 days) [78], and their high dose concentrations (between 70 and 140 mg/ml) should be of specific interest to researchers when considering how low physiological levels of CGRP are in the human blood stream (5–10 × 10^−6^ µg/ml) [79]. CGRP concentration in the plasma is very low. This low concentration is partly due to the nature of the communication within the nervous system where only small concentrations of a certain protein are needed at a location, assisted by the vast reach of nerve endings and, to an important degree, the potency neuropeptides possess in general. Therefore, it is of great concern that, according to the Public health-European commission’s (EUROPA) report discussing the pharmacokinetic properties of erenumab, ‘subcutaneous administration of a 140 mg once monthly dose and a 70 mg once monthly dose in healthy volunteers resulted in a Cmax mean (maximum serum concentration) (standard deviation [SD]) of 15.8 (4.8) μg/ml and 6.1 (2.1) μg/ml’ (https://ec.europa.eu/health/documents/community-register/2018/20180726141585/anx_141585_en.pdf). This statement indicates that the concentration of erenumab in the plasma after treatment is 10^6^ times higher than that of CGRP levels in the plasma, even when CGRP plasma levels elevate during a migraine attack to aprox 80 pmol/l (20 pmol/l at rest) [19] this concentration remain substantially high. Systemically blocking CGRP function to this extent, with little regard to its many roles in other biological cascades (i.e., its critical attributes in shaping the immune outcomes of disease), is of concern. The findings in the EUROPA report correlated with the findings in the report from the European Medicines Agency (EMA).

Of note, it is important to emphasize that the thorough examination of CGRP and the immune response in this review must not overshadow the roles of CGRP in the neuronal milieu, namely, countering vasoconstrictive neuropeptides, nociception processes and nerve regeneration and health, which are all important topics that only add further validation to raised concerns. Only last month, concerns were forwarded regarding the vasoconstrictive reactions of these drugs [80], in which patients exhibited severe blood flow constriction (patients developed Raynaud’s phenomenon) after treatment with 3 of the 4 mentioned CGRP/CGRPR blockers. Moreover, toxicity studies have been conducted on CGRP Blockers, [81, 82]. Findings are noteworthy, however, they do not discuss the effects of these drugs on underlying immunological challenges, which is the focus of this review. These studies widen the scope of investigations into potential adverse effects outside of the neuro-immune context, which would possess their own merit if investigated fairly.

Regarding the environmental impact, efficient background investigations may prevent an unplanned increase/epidemic of treatable infections and physiological disturbances with the penetration of the new products into market, which is likely to be rapid due to the number of sufferers of migraine. This will be particularly challenging if these drugs are released into developing countries where over the counter availability could fuel a loss of control, as it has with antibiotic resistance, especially in Africa and Asia where parasitic infections preside. Studies on the socioeconomic impact of these drugs are emerging [83, 84]. While only limited to first world countries, these studies are a valuable inclusion to our understanding of the environmental impacts of CGRP/CGRPR inhibitors. However, similar studies accompanying the penetration of these drugs into the markets of third world countries is required due to the nature of relaxed regulations of drug distribution and prescriptions compared to other markets around the globe.

Of course, some may ask: can there be a different angle to approach migraines other than CGRP? Interestingly, the release of CGRP is downstream of the neuronal cascade thought to be activated during migraines, and thus, it might be possible to avoid the widespread side effects of interfering with CGRP by targeting the upstream process specific to migraines. The activation of the trigeminal-vascular system results in the release of a number of vasoactive neuropeptides, including CGRP and substance P [85]. Experimental activation of trigeminal ganglion cells results in the release of CGRP, which is inhibited by 5-HT_1D/1B_ agonists in a dose-dependent manner, highlighting the role of serotonin in regulating CGRP-induced vasodilation [86, 87]. Therefore, this manuscript also presents an alternative opinion to the current. Finally, it is important to note that this is not the first attempt at raising concerns related to the new wave of migraine treatments [88, 89]. In fact, just recently, several studies have emerged, only 10 months after erenumab was approved questioning its safety. A very passionate appeal was made by a leading physician (Lawrence Robbins, MD) argued that it is too early to label erenumab ‘very safe’ due to the increased numbers of reports listed on the FDA ‘Adverse Events Reporting System Public’ Dashboard. In total, 10,531 adverse events related to erenumab were recorded by July 2019 [90]. In his letter, Dr. Robbins stated that in only 10 months, of the 10,531 adverse events recorded, 1460 events were considered serious, and some even life threatening. This statement follows a previous letter by the same author the previous year warning of potential long-term adverse effects [91]. Additionally, a medical brief highlighted the hypersensitivity reactions that have been associated with anti-CGRP/CGRPR treatments, while reiterating the need to determine the consequences and severity of these reactions [92]. Following these appeals, this review finds grounds to further discuss the risks associated with these drugs.

In summary, this review concludes that sufferers of chronic migraine treated with anti-CGRP/CGRPR drugs having underlying immunological challenges or/and are under threat of new pathological insults, are the most susceptible to potential adverse effects. Understandably, cautious and mindful approaches when using CGRP/CGRPR blockers, regardless to the model and reasoning behind its administration, are to be considered. Proposed measures including i) closely monitoring patients treated with anti-CGRP/CGRPR drugs, especially those prone to certain viral, bacterial and parasitic infections, and ii) the use of tailored dosages, in a gradual dose vs. symptom alleviation-dependent manner are strongly recommended.

## Data Availability

Not applicable.

## Abbreviations

ADCC: Antibody-dependent cytotoxicity
CGRP: Calcitonin gene related peptide
CGRPR: CGRP receptor
CRLR: Calcitonin receptor-like receptor
CXCR4: C-X-C chemokine receptor type 4
CXCL8: C-X-C chemokine motif type 8
EMA: European Medicines Agency
EU EPAR: European public assessment reports
EUROPA: Public health-European commission
GDNG: Glial cell line-derived neurotropic factor
HNP-1: Alpha-defensin
IFN: Interferon
LC: Langerhans cells
LL37: Cathelicidin
LPS: Lipopolysaccharide
NGF: Nerve growth factor
RAMP1: Receptor activity-modifying protein 1
SmPC: European summary of product characteristics
Th: T lymphocyte helper cells
TNF: Tumour necrosis factor
TRPV1: Transient receptor potential vanilloid 1
USPI: United States prescribing information
VZV: Varicella zoster virusIntroduction
