# Correction to: Anti-migraine agents from an immunological point of view

**DOI:** 10.1186/s12967-021-02988-y

**Published:** 2021-08-09

**Authors:** Mushref Bakri Assas

**Affiliations:** Faculty of Applied Medical Sciences, Department of Medical Laboratory Technology, Immunology Group, King Abdul Aziz University, Jeddah, Saudi Arabia

## Correction to: J Transl Med (2021) 19:23 10.1186/s12967-020-02681-6

Following publication of the original article [1], the author identified an error in the author name of **Mushref Bakri Assas**.

- The incorrect author name is: Bakri M. Assas
- The correct author name is: Mushref Bakri Assas

The author group has been updated above and the original article [1] has been corrected.
